# Urinary glycosaminoglycan excretion in patients with primary nocturnal enuresis

**DOI:** 10.1186/1824-7288-36-13

**Published:** 2010-02-03

**Authors:** Yasemin U Budak, Kağan Huysal, Atilla Guray

**Affiliations:** 1 Department of Clinical Chemistry and Laboratory Medicine, Sevket Yilmaz Hospital, Bursa, Turkey; 2 Department of Clinical Chemistry and Laboratory Medicine, Yuksek Ihtisas Training Hospital, Bursa, Turkey; 3 Department of Pediatrics, Sevket Yilmaz Hospital, Bursa, Turkey

## Abstract

**Aim:**

The aim of this study was to determine whether primary nocturnal enuresis (PNE) leads to alterations in glycosaminoglycan (GAG) excretion.

**Methods:**

Twenty subjects (mean age 8.7 years, M/F 15/5) with PNE were included in the study. Twenty-two healthy subjects were selected as a control group (mean age 8.7 years, M/F 14/8). Urinary GAG excretion was measured using a modified dimethylmethylene blue (DMD) assay of 24-hour urine.

**Results:**

The mean urinary GAG content was 33.9 mg/g and 23.8 mg/g creatinine in patients with PNE and controls, respectively; patients with PNE thus had a higher mean GAG excretion than did age-matched controls (p < 0.05). The association between GAG level and enuresis frequency bordered on significance (p = 0.068).

**Conclusions:**

GAG excretion in patients with PNE was significantly higher than in normal children, suggesting that measurement of urinary GAG may be useful in evaluating physiopathological conditions of the bladder wall. Further studies are needed to confirm this finding.

## Background

Nocturnal enuresis (NE) is defined as repeated, spontaneous voiding of urine during sleep in a child aged 5 years or older [1]. Available evidence indicates that NE is best regarded as a group of conditions with different etiologies rather than a single entity, because only a multifactorial etiology can explain the wide range in outcomes and the fact that different patients respond to different therapies [2]. NE may be classified as primary or secondary [3]. Primary nocturnal enuresis is bedwetting in a child aged 5 years or over who has never been dry for extended periods, whereas secondary nocturnal enuresis is the onset of wetting after a continuous dry period of more than 6-12 months [4]. Primary nocturnal enuresis (PNE) is one of the most frequent complaints in pediatric andurologic practice. PNE is caused by a disparity between bladder capacity and nocturnal urine production, and failure of the child to awaken in response to a full bladder [5]. Despite numerous studies on PNE, the etiology remains elusive.

Recent studies have focused on the importance of glycosaminoglycans (GAGs; linear polysaccharide chains composed of dimers of a couplet consisting of hexuronic acid-amino sugar) that line the transitional epithelium of the human bladder. GAGs play a structural role in the extracellular matrix, regulate ion permeability, and have anti-inflammatory functions on the bladder surface and wall [6-8]. The aim of the present study was to investigate variation in GAG excretion in patients with PNE.

## Patients and Methods

The study included 20 children with PNE (15 boys and 5 girls, mean age 8.7 years, range 6-14 years) and 22 healthy gender- and age-matched controls.

Exclusion criteria were conditions associated with large urine volume, abnormal neurological control, daytime wetting, and previous use of prescription medications for enuresis. Urinary symptoms were evaluated by taking of a history from each affected child and the parents, and by use of a questionnaire and bladder diary. All children underwent urinalysis. No child had any evidence of urinary tract infection (UTI). Exclusion of UTI was based both on urinalysis and absence of symptoms including dysuria, urination frequency or urgency, fever, or costolumbar or suprapubic pain. Pyuria, nitrite in the urine, leukocyte esterase activity (LEA) in the urine, or positive urine culture (>10^5 ^CFU/mL), were all considered evidence of UTI [9].

A dipstick test confirmed no abnormalities in pH or urine specific gravity, and the absence of protein, glucose, ketones, urobilinogen, bilirubin, or blood. All children had normal renal function, as determined by blood urea and serum creatinine measurements.

Urinary glycosaminoglycan excretion was spectrophotometrically determined in urine samples (at 520 nm) after addition of dimethylmethylene blue (Sigma-Aldrich, St. Louis, MO) using bovine kidney heparan sulfate as a standard (Sigma; catalog no. H 7640) [10]. Under our experimental conditions the intra- and inter-assay coefficients of variation were 1.5% and 2.4% for 10 mg/L glycosaminoglycan, respectively. The GAG results were expressed as a GAG/creatinine (mg/g, respectively) ratio.

All values are presented as means ± SDs. Statistical analysis employed SPSS, version 10.0 (SPSS, Inc, Chicago, IL). Statistically significant differences between parameters were established using Student's unpaired t test. Pearson correlations were used to determine the relationship between bedwetting frequency and urinary GAG level. A p value <0.05 was considered to indicate a significant difference.

## Results

**Figure 1:**
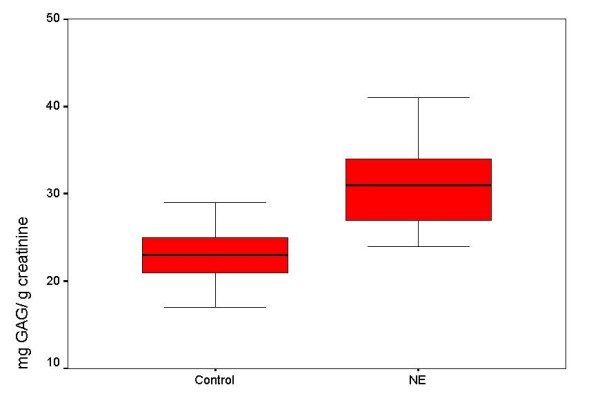
**Mean (SD) urinary GAG/creatinine ratio (mg/g) in controls and in patients with primary nocturnal enuresis**.

Of the 20 children currently bedwetting, 10 children had nocturnal enuresis every night, 4 at least three times a week, and 6 once or twice a week (Table 1). The mean urinary GAG content was 33.9 ± 9.3 mg/g, and 23.8 ± 6.2 mg/g creatinine, in patients with PNE and controls, respectively; patients with NE thus showed a higher mean GAG excretion than did age-matched controls (p < 0.05) (Figure 1).

**Table 1:** Bedwetting Frequency

Frequency	Boys n (%)	Girls n (%)
Every night	9 (45)	1 (5)
3> nights/week	2 (10)	2 (10)
<3 nights/wk	3 (15)	3 (15)

Overall, the correlation between bedwetting frequency and urinary GAG level bordered on significance (r = 0.415, p = 0.068). We consider it likely that future work with larger numbers of patients would show a significant correlation between GAG level and enuresis frequency.

## Discussion

All cells apparently have GAGs on the surface, where the molecules constitute a matrix. Because of the negative charge of sulfate, GAGs repel each other, forming a type of cushion effect when cells interact. GAGs are very important in directing traffic at the surface, and constitute a necessary holding zone for many chemicals that must mutually associate at the cell surface. GAGs prevent small molecules from reaching the underlying tight junctions and cell membranes, and, hence, form a major permeability barrier [11]. Several reports on the functional role of GAGs in the urinary tract have appeared. GAGs are an obligatory constituent of the basal lamina of the urothelium, contributing to maintenance of transmembrane permeability and preventing stone formation [12,13]. The presence of GAGs on the bladder wall surface is necessary for inhibition of bacterial adherence and infection, and to avoid tumor cell implantation [14]. GAGs excreted in the urine of healthy subjects or of patients with urological disease (*e.g.*, interstitial cystitis or spina bifida) are probably derived from damaged bladder epithelium. Recently, Salvaggio and colleagues found that increased excretion of GAGs in spina bifida patients was an important parameter in evaluation of the physiopathological condition of the bladder wall [15]. The cited authors found that the wall became rich in GAG in patients with neurogenic bladders, leading to wall degeneration. Thus, it was suggested that urinary GAG excretion could be a valuable marker indicating commencement of bladder damage.

Interstitial cystitis is often treated with exogenous GAGs such as heparin, chondroitin sulfate, hyaluronate, or semi-synthetic polysulfate. The mechanism of action is presumed to be coating of the bladder surface to replace chondroitin sulfate and heparin sulfate lost as a result of the disease.

In the present study, GAG excretion was higher in patients with PNE than in healthy controls. Similarly, Ferrara and colleagues found that urinary GAG excretion was significantly elevated in patients with isolated NE compared to controls [16]. They compared GAG excretion in 15 patients with isolated NE, and 12 NE patients who also suffered from diurnal incontinence, with GAG excretion in 27 healthy age-matched controls. The cited authors suggested that measurement of GAG excretion in such patients might be useful for evaluation of the physiopathological condition of the bladder wall, and hence in the monitoring of potential damage to the bladder mucosa.

Caione and associates investigated the effectiveness of a dextranomer/hyaluronic acid copolymer in treatment of urinary incontinence caused by sphincter incompetence in a total of 16 children and adolescents. At 6, 12, and 24 months of follow-up, patients reported improvement in daytime and nighttime dryness, respectively [17].

Increased GAG excretion might result from either immaturity of bladder innervation, or chronic exfoliation of the bladder surface in response to infection. We suggest that quantitative and/or qualitative defects in the GAG layer might lead to reduction in functional bladder capacity.

Although the association between bedwetting frequency and urinary GAG level only bordered on significance, analysis of a larger patient series might permit a stastically significant association between these variables to be demonstrated.

Despite our small sample size, the between-group difference in urinary GAG values attained statistical significance, and we thus suggest that measurement of urinary GAG may be useful in evaluating physiopathological conditions of the bladder wall. Further studies are needed to confirm the validity of this observation.

## Competing interests

The authors declare that they have no competing interests.

## Authors' contributions

All authors have made substantial contributions to design of the work, in addition to analysis and interpretation of data; and have been involved in drafting the article and revising it critically for important intellectual content; and have given final approval of the version to be published.
